# The Kinetochore Is an Enhancer of Pericentric Cohesin Binding

**DOI:** 10.1371/journal.pbio.0020260

**Published:** 2004-07-27

**Authors:** Stewart A Weber, Jennifer L Gerton, Joan E Polancic, Joseph L DeRisi, Douglas Koshland, Paul C Megee

**Affiliations:** **1**Department of Biochemistry and Molecular Genetics, University of Colorado Health Sciences Center at FitzsimonsAurora, Colorado, United States of America; **2**Stowers Institute, Kansas CityMissouri, United States of America; **3**Department of Biochemistry and Biophysics, University of CaliforniaSan Francisco, California, United States of America; **4**Howard Hughes Medical Institute, Department of EmbryologyCarnegie Institution of Washington, Baltimore, MarylandUnited States of America

## Abstract

The recruitment of cohesins to pericentric chromatin in some organisms appears to require heterochromatin associated with repetitive DNA. However, neocentromeres and budding yeast centromeres lack flanking repetitive DNA, indicating that cohesin recruitment occurs through an alternative pathway. Here, we demonstrate that all budding yeast chromosomes assemble cohesin domains that extend over 20–50 kb of unique pericentric sequences flanking the conserved 120-bp centromeric DNA. The assembly of these cohesin domains requires the presence of a functional kinetochore in every cell cycle. A similar enhancement of cohesin binding was also observed in regions flanking an ectopic centromere. At both endogenous and ectopic locations, the centromeric enhancer amplified the inherent levels of cohesin binding that are unique to each region. Thus, kinetochores are enhancers of cohesin association that act over tens of kilobases to assemble pericentric cohesin domains. These domains are larger than the pericentric regions stretched by microtubule attachments, and thus are likely to counter microtubule-dependent forces. Kinetochores mediate two essential segregation functions: chromosome movement through microtubule attachment and biorientation of sister chromatids through the recruitment of high levels of cohesin to pericentric regions. We suggest that the coordination of chromosome movement and biorientation makes the kinetochore an autonomous segregation unit.

## Abstract 2

Kinetochores mediate chromosome movement through microtubule attachment and biorientation of sister chromatids through the recruitment of high levels of cohesin to pericentric regions

## Introduction

The proper segregation of replicated chromosomes, or sister chromatids, to daughter cells during mitosis requires that sister chromatids establish stable attachments to microtubules emanating from opposite spindle poles, known as chromosome biorientation, and that sister chromatids move to opposite poles of the cell during anaphase. Chromosome biorientation is made possible by the cohesion of replicated sister chromatids, which occurs along the entire length of the chromosome and is especially robust in large centromere-flanking or “pericentric” domains (Sumner 1991). The centromere is the site of assembly of the kinetochore, a protein complex that mediates the attachment and movement of chromosomes along the mitotic spindle. The centromere-flanking domains of cohesion are thought to play an important role in biorientation by constraining the kinetochores on paired sister chromatids in opposite directions. This orientation of sister kinetochores facilitates the capture of microtubules originating from different spindle poles, thereby ensuring that sister chromatids are segregated in opposition later in mitosis. A second function of pericentric cohesion and possibly cohesion along chromosome arms is to resist the poleward forces that are imposed by these bipolar spindle microtubule attachments. The resistance that is provided by cohesion prevents the premature dissociation of sister chromatids and contributes to a tension-based mechanism that stabilizes kinetochore–microtubule interactions (Nicklas and Ward 1994).

Given the importance of cohesion in pericentric regions, it is critical to understand how these large functional domains are assembled. An important clue came with the discovery of a group of proteins that mediate cohesion and are conserved in organisms from the yeasts to vertebrates (Guacci et al. 1997; Michaelis et al. 1997; Furuya et al. 1998; Losada et al. 1998; Skibbens et al. 1999; Toth et al. 1999; Hartman et al. 2000; Tomonaga et al. 2000; Wang et al. 2000; Hanna et al. 2001; Losada and Hirano 2001). This group includes Pds5p and the members of a multisubunit “cohesin” complex, Mcd1/Scc1p, Irr1p/Scc3p, Smc1p, and Smc3p. Cohesins were shown to bind to specific regions of chromosomes (Blat and Kleckner 1999; Megee et al. 1999; Tanaka et al. 1999; Hartman et al. 2000; Laloraya et al. 2000; Panizza et al. 2000). At centromere-distal locations, cohesin-binding sites span only approximately 0.8 kb (Laloraya et al. 2000). In striking contrast, cohesin binding is highly enriched within an approximately 50-kb pericentric region of budding yeast Chromosome (CHR) III flanking the centromere–kinetochore complex, which occupies only a 0.25-kb nuclease-resistant region (Bloom and Carbon 1982; Saunders et al. 1988; Blat and Kleckner 1999). It remains to be determined whether pericentric cohesin enrichment is unique to budding yeast CHRIII or is, in fact, a property of all pericentric regions. However, cytological observations support the notion that cohesin may be enriched in the centromere-proximal regions of all higher eukaryotic chromosomes, given that the chromosomes of mitotically arrested cells remain tightly associated in these regions (Gonzalez et al. 1991). These observations suggest that these large domains of pericentric cohesion result from an enrichment of cohesin binding.

How are these large cohesin domains assembled in kinetochore-flanking regions? Recent experiments in Schizosaccharomyces pombe have provided evidence for the role of repetitive heterochromatic DNA in pericentric cohesion. In this organism, the recruitment of high levels of cohesion factors to centromeric regions is dependent on Swi6 (SPAC664.01c), the fission yeast homolog of the heterochromatin protein HP1 (Bernard et al. 2001; Nonaka et al. 2002). However, this is unlikely to be the only mechanism for generating large pericentric cohesin domains because the centromere on budding yeast CHRIII and neocentromeres in human cells are devoid of surrounding repetitive sequences characteristic of heterochromatin. One clue for a potential mechanism came from our observation that *CEN3* placed ectopically on a minichromosome could direct the binding of cohesin to approximately 2 kb of centromere-flanking DNA even if that DNA did not normally associate with cohesin, establishing that centromeres could modulate cohesin binding on neighboring sequences (Megee et al. 1999).

The presence of these cohesin-enriched domains flanking *CEN3* on both the endogenous chromosome and a minichromosome is intriguing, and their existence has raised many interesting questions. Here, we endeavored to determine whether large pericentric cohesin domains exist on all budding yeast chromosomes, and if so, whether these domains are equivalent in size and have a similar distribution of cohesin. Furthermore, we examined the possible roles of the centromere and centromere-flanking sequences in the assembly of the pericentric cohesin domains. Our results show that the budding yeast kinetochore behaves as an enhancer in the assembly of approximately 20-kb to 50-kb pericentric cohesin domains. Our observations suggest that the kinetochore mediates two essential segregation functions, coordinating not only the attachment of chromosomes to the mitotic spindle, but also the recruitment of sufficient levels of cohesin within pericentric regions to promote biorientation of sister kinetochores and to resist microtubule-dependent poleward forces. Thus, the kinetochore functions as a modular segregation unit. The integration of these functions by the kinetochore is therefore likely to play an important role in the maintenance of genomic integrity.

## Results

### Cohesin Is Enriched throughout Large Pericentric Domains Relative to Arm Sites

Previous studies have demonstrated that cohesin binding is highly enriched in the centromere-proximal region of CHRIII in comparison to arm sites and also within the centromere-flanking region of a *CEN3*-containing minichromosome in budding yeast arrested in mitosis using nocodazole, an inhibitor of microtubule assembly (Blat and Kleckner 1999; Megee et al. 1999; Laloraya et al. 2000). To examine cohesin binding throughout centromere-proximal and -distal regions of the budding yeast genome, we have performed chromatin immunoprecipitation (ChIP) using epitope-tagged alleles of cohesin subunits (Mcd1-6HAp or Smc3-6Mycp) as markers for the cohesin complex. The immunoprecipitated (ChIP) and input DNA samples not subject to immunoprecipitation were then analyzed using two approaches (Materials and Methods). In some cases, PCR reactions that respond linearly to the amount of input DNA were performed for both ChIP DNA and diluted input DNA to determine the percentage of total chromatin bound by cohesin subunits in the ChIPs. Alternatively, input and ChIP DNA samples were labeled with aminoallyl dUTP conjugated to different fluorescent tags, and then hybridized competitively to DNA microarrays containing all budding yeast open reading frames (ORFs) and intergenic regions to analyze cohesin binding genome-wide. In both types of experiments, we examined the distribution (i.e., the locations of peaks and valleys) and the magnitude (peak height) of cohesin subunit binding.

**Figure 1:**
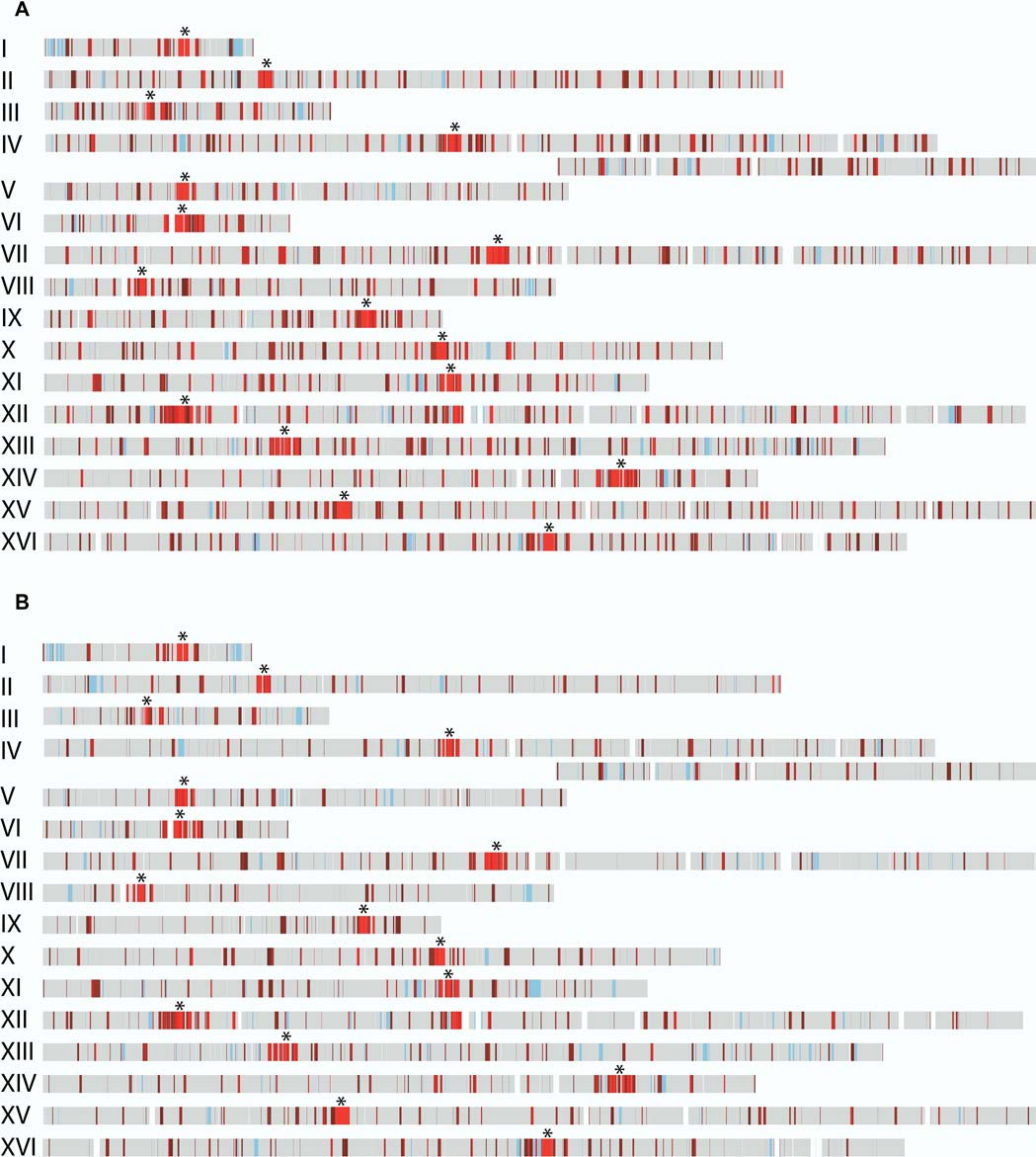
Microarray Analyses of Mcd1p and Smc3p Binding DNA isolated from cohesin subunit ChIPs and control input DNA was labeled with aminoallyl dUTP, conjugated to Cy5 (red) or Cy3 (green) fluorescent tags, and then hybridized competitively to microarrays. Although the samples were labeled by different fluorescent tags depending on the experiment, for the purposes of analysis, the ratios are converted such that the ChIP signal is represented by red and control DNA by green. The red-to-green (R:G) ratio for each ORF and intergenic region was calculated for cells arrested in mitosis using the *cdc16* mutation and assigned a color, and the median value obtained for each element was then plotted on a map of the sixteen chromosomes to determine the chromosomal distribution of Mcd1–6HAp. Regions with a R:G ratio less than 1.8 are shown in gray, and those with ratios of 1.8 or higher are shown in red. The red shading is an indicator of the intensity of cohesin subunit binding, such that regions with larger R:G ratios have lighter shades of red. Hybridization data are unavailable for regions shaded in blue (see Materials and Methods), and genomic regions not present on the arrays are indicated in white. Centromere position is indicated by an asterisk. (A) Chromatin isolated from strains containing Mcd1-6HAp (1377A1-4B, 1829-15B, and PMY270) was immunoprecipitated using anti-HA antibodies. (B) Chromatin isolated from strains containing Smc3-6Mycp (PMY270 and 1839-3D) was immunoprecipitated with anti-Myc antibodies.

**Figure 2:**
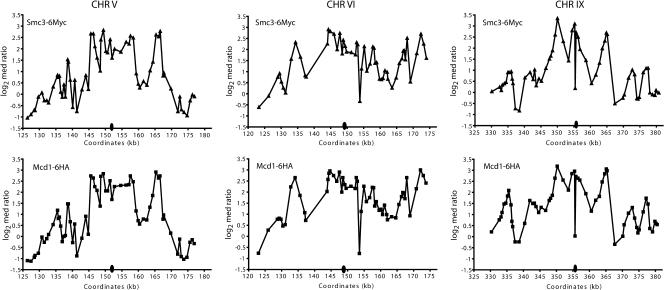
Comparison of Mcd1p and Smc3p Binding Distributions in Centromere-Flanking Regions The log_2_ of the median of the R:G ratios for Smc3-6Mycp (triangles) and Mcd1-6HAp (squares) binding within approximately 60-kb pericentric regions of CHRV, CHRVI, and CHRIX in *cdc16*-arrested cells is plotted as a function of the indicated SGD coordinates. The relative position of the centromere within each pericentric region is indicated by the oval.

To determine whether the pericentric regions of all budding yeast chromosomes are similarly enriched for cohesin binding, input DNA and DNA crosslinked to Mcd1p in mitotically arrested cells were used to probe microarrays of the budding yeast genome. The profile of Mcd1p binding within each ORF and intergenic region was then superimposed on a map of the sixteen budding yeast chromosomes to visualize the distribution of Mcd1p association (Figure 1A). This analysis demonstrated dramatic differences in the distribution of Mcd1p in centromere-proximal and -distal regions of all sixteen budding yeast chromosomes, as had been observed previously in a study of CHRIII (Blat and Kleckner 1999). While centromere-distal regions contained short, discrete foci (approximately 0.8 kb) of Mcd1p binding that were distributed at intervals of approximately 10 kb, centromere-proximal regions of all chromosomes contained large (approximately 20–50 kb) domains that were highly enriched for Mcd1p binding. Microarray analyses were also performed using ChIP DNA isolated from strains containing both Myc-tagged Smc3p (Smc3-6Mycp) and HA-tagged Mcd1p (Mcd1-6HAp), or Smc3-6Myc alone to confirm that another subunit of the cohesin complex showed a similar enrichment in pericentric chromatin. As was the case with Mcd1p, the magnitude of Smc3p association was higher in pericentric regions than at arm locations (Figure 1B). Furthermore, the pattern of Smc3p binding observed within pericentric regions in independent immunoprecipitations of chromatin isolated from the singly or doubly tagged strains was strikingly similar to that observed for Mcd1p (Figure 2). Thus, the high level of concurrence in cohesin subunit binding patterns suggests that the association of the entire cohesin complex is enriched in pericentric chromatin compared to chromosome arm locations. In addition, we compared the datasets obtained by microarray analyses in this study to those obtained using Myc-tagged Mcd1p (Mcd1-18Mycp) in another commonly used budding yeast strain background as described in the accompanying report by Glynn et al. (2004). These studies showed good agreement for the presence of enriched Mcd1p binding in pericentric regions and a similar distribution of peaks and valleys of binding at both pericentric and arm locations (correlation coefficient = 0.76; Glynn et al. 2004).

**Figure 3:**
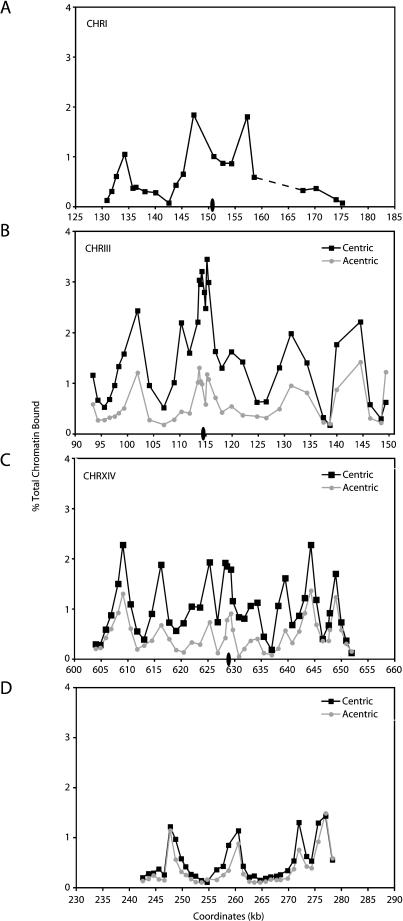
Mcd1p Binding Profiles in Centromere-Proximal and -Distal Regions Cells containing Mcd1-6HAp were first staged in G1 using αF, and then released from G1 into medium containing nocodazole to arrest the cells in mitosis. For the centromere excision experiments (B–D), the cultures were divided in half after G1 arrest, and one half of each culture was treated with galactose for 2 h to induce centromere excision (see Materials and Methods). Both the induced (acentric) and uninduced (centric) control cultures were then released from the G1 arrest into fresh medium and rearrested in mitosis. Once arrested in mitosis, cells were fixed in formaldehyde and then processed for ChIP using antiserum against epitope-tagged Mcd1p (Mcd1-6HAp) as an indicator of the cohesin complex. DNA isolated from the ChIPs and diluted input DNA not subject to immunoprecipitation were then subjected to PCR analysis using oligonucleotide primer pairs that amplify approximately 300-bp fragments within the indicated regions. Quantitation of DNA in the Mcd1p ChIPs, expressed as a percentage of the input DNA, is plotted as a function of the locations of the midpoints of those DNA fragments based on the SGD coordinates. Centromere position is indicated by an oval (not drawn to scale). (A) The Mcd1p association profile for the CHRI pericentric region in strain 1377A1-4B is shown. Mcd1p binding adjacent to *CEN1* is difficult to assess fully because of the presence of a moderately repetitive Ty element in the region from approximately 160 to 166 kb, indicated with the dashed line. Similarly, the Mcd1p binding profiles in the pericentric regions of CHRIII (B) and CHRXIV (C) are shown in the presence (black squares) and absence (gray circles) of *CEN3* and *CEN14* using strains PMY185 and PMY206, respectively. (D) The Mcd1p binding profiles for a centromere-distal region of CHRIII are shown for comparison in the presence (black squares) and absence (gray circles) of *CEN3*.

**Figure 4:**
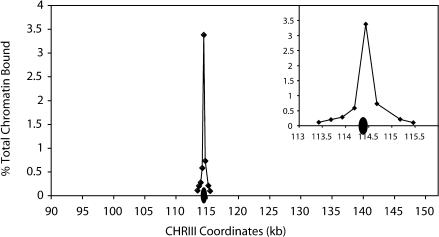
Shearing of Centromere-Proximal Chromatin by Sonication As a control for the shearing of chromatin, a precipitation of chromatin was performed in each experiment using a polyclonal antiserum specific for the kinetochore protein Mif2p, which has been shown to interact with centromeric DNA (Meluh and Koshland 1997). DNA crosslinked to Mif2 was then subjected to PCR analysis throughout a 2-kb region spanning *CEN3,* using a series of primer pairs that amplify 240 ± 21–bp fragments. *CEN3* DNA spans SGD coordinates 114382 to 114498, indicated with the ovals. Data were plotted on a scale similar to cohesin subunit ChIP data for comparison, and the inset shows in detail the magnitude of binding within a 2-kb centromere-flanking region.

To further investigate the nature of this pericentric cohesin enrichment, Mcd1p association profiles were examined within the approximately 50-kb pericentric regions of endogenous CHRI, CHRIII and CHRXIV using PCR analyses of ChIP DNA. For comparison, Mcd1p association was also examined within an approximately 37-kb centromere-distal region on the right arm of CHRIII (*Saccharomyces* Genome Database [SGD] coordinates 242–279 kb) (Figure 3). We observed that the magnitude of Mcd1p binding throughout these pericentric regions is on average 3- to 5-fold higher than the levels of association observed at the CHRIII centromere-distal location. Within each of the pericentric domains examined, the distribution of Mcd1p binding was not uniform, but instead consisted of peaks and troughs of association (Figure 3A–3C). These peaks of Mcd1p binding were much broader than those observed at the CHRIII centromere-distal location (Figure 3D) (Blat and Kleckner 1999; Laloraya et al. 2000). The peaks of Mcd1p association within pericentric chromatin were separated by troughs having values approximately 0.5% of those of the input chromatin. Although significantly reduced in comparison to the peaks, Mcd1p association in these troughs reflects significant binding, given that other chromosomal regions such as *ARS1* and *ADE3* are absent from Mcd1p ChIPs (≤0.04% of input chromatin; unpublished data not shown). Furthermore, this binding within troughs is unlikely to reflect poor shearing of the pericentric chromatin by sonication, as ChIPs performed using antibodies specific for the kinetochore protein Mif2p showed an enrichment within centromeric DNA that decreased 5-fold in flanking regions only approximately 245 bp away from *CEN3* (Figure 4). Lastly, it is interesting to note that in many of the pericentric regions, cohesin subunit association in the interval that contains the centromeric DNA was reduced in comparison to the regions immediately flanking the centromere, possibly because the large complex of kinetochore proteins precludes the association of cohesin with the relatively small 120-bp centromeric DNA (see Figures 2 and 3A–3C). Thus, microarray and PCR analyses of DNA crosslinked to cohesin subunits demonstrate that cohesin binding is highly enriched within large pericentric domains on all budding yeast chromosomes.

While all pericentric regions were enriched for Mcd1p and Smc3p binding, we found that the pattern of cohesin subunit association was different within each pericentric region (Figure 3A–3C). For example, the CHRIII pericentric region had its highest levels of Mcd1p association within an approximately 2-kb region spanning the centromere, and this region was flanked symmetrically by peaks of Mcd1p association of lesser magnitude, located approximately 15 kb from the centromere. In contrast, the CHRI centromere was located in a local trough of Mcd1p association and was flanked by peaks of Mcd1p binding that were approximately 5 kb away. Furthermore, the pericentric region of CHRXIV contained a series of closely spaced peaks of Mcd1p association that were roughly equivalent in magnitude. Thus, although all pericentric regions were indeed enriched for cohesin association, the uniqueness of the distribution of cohesin within each pericentric region suggests that cohesin binding is influenced by local sequence characteristics.

### Enhancement of Pericentric Cohesin Binding Is Mediated by the Kinetochore

**Figure 5:**
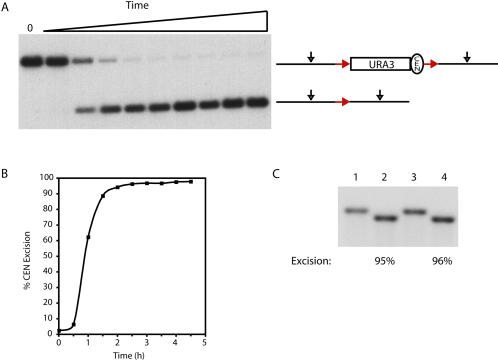
Centromere Excision (A) *CEN1* on CHRI was replaced with a *CEN3-URA3* cassette flanked by head-to-tail-oriented site-specific recombination target sites (red arrows) for the R recombinase from *Zygosaccharomyces rouxii,* as described in Materials and Methods. This strain (1824-23B) contained the R recombinase under the control of a galactose-inducible promoter. Genomic DNA samples, taken prior to the addition of galactose to the culture medium (0) and at 0.5-h intervals for 4.5 h after the galactose addition, were digested to completion with PvuII (black arrows) and analyzed by Southern blot analysis using a 1.25-kb probe corresponding to CHRI SGD coordinates 151823 to 153080. (B) The percentage of centromere excision was determined for the timecourse shown in (A). Briefly, a phosphorimage of the Southern blot and ImageQuant software were used to determine the pixel intensities of the unexcised and excised bands (top and bottom bands, respectively). The percent excision was then calculated as the pixel intensity present in the excised band divided by the total pixel intensities of both bands at each timepoint. (C) A Southern blot analysis of centromere excision from CHRIII. The endogenous *CEN3* on CHRIII was replaced by R-recombinase target-site-flanked *CEN3* in strain 1829-15B, as described in Materials and Methods. The efficiency of centromere excision from CHRIII was determined by Southern blot analysis in two independent experiments using genomic DNA samples digested with SnaBI and a probe corresponding to CHRIII SGD coordinates 113799-114336. Lanes 1 and 3 represent uninduced controls, and lanes 2 and 4 represent the extent of centromere excision after 2 h of recombinase induction. The percent excision was determined as in (B).

Although pericentric cohesin recruitment in S. pombe likely occurs through an interaction with the heterochromatin constituent HP1 bound to repetitive DNA sequences, the absence of repetitive DNA flanking budding yeast centromeres indicates that some other mechanism is used for the recruitment of cohesin throughout large pericentric domains. One possibility is that the high density of cohesin within pericentric chromatin is dependent on the centromere–kinetochore complex. To test this possibility, we examined Mcd1p association in the centromere-flanking regions of chromosomes in the presence and absence of the centromere. For these experiments, the endogenous centromeres on CHRIII and CHRXIV were replaced by *CEN3* and *CEN14* sequences, respectively, flanked by site-specific recombination target sites for the R recombinase from Zygosaccharomyces rouxii (Materials and Methods). The 120-bp centromeric DNA and approximately 200 bp of flanking sequences, corresponding roughly to the nuclease-resistant region spanning budding yeast centromeres (Bloom and Carbon 1982), were then excised from the chromosome in G1 cells by activating the expression of a galactose-inducible R recombinase, resulting in the generation of an acentric chromosome. The absence of Mcd1 protein in G1-staged cells and the interdependency of cohesin subunit association with chromosomes suggest that centromere excision occurs prior to the loading of the cohesin complex onto chromosomes in our experimental regimen (Guacci et al. 1997; Toth et al. 1999). After centromere excision (≥95% efficiency; Figure 5), the cells were released from the G1 arrest, rearrested in mitosis using nocodazole, and then processed for ChIP to assess Mcd1p binding.

In nocodazole-arrested cells, the magnitude of Mcd1p binding within the approximately 50-kb region flanking the site of the excised *CEN3* was reduced significantly compared to control cells that retained the centromere (see Figure 3B). This reduction in Mcd1p binding occurred symmetrically throughout the entire 50-kb pericentric region flanking *CEN3*. In contrast, the magnitude of Mcd1p binding within a centromere-distal location on the arm of CHRIII was similar in both the centric and acentric chromosomes, indicating that cohesin association on chromosome arms is unaffected by centromere excision (see Figure 3D). Furthermore, the magnitude of Mcd1p association within the pericentric region of an endogenous chromosome (CHRI) that did not undergo centromere excision was also unaltered (unpublished data). In agreement with the results for CHRIII, we also observed a symmetrical reduction in Mcd1p binding throughout an approximately 50-kb pericentric region on an acentric CHRXIV when compared to control cells that retained *CEN14* (see Figure 3C). While the magnitude of Mcd1p association was dramatically reduced in the former pericentric regions following centromere excision, the relative positions of the peaks and troughs of Mcd1p binding were unaltered. These results suggest that the centromere is required for the enrichment of Mcd1p binding in pericentric regions, where it amplifies an intrinsic local pattern of Mcd1p association. This amplification appears to occur bidirectionally, even though the DNA and protein components of the centromere–kinetochore complex are inherently asymmetric (Espelin et al. 1997). In addition, the loss of pericentric cohesin binding upon centromere excision occurred despite the presence of centromeres on the remaining chromosomes. Thus, the enhancement of cohesin binding in pericentric chromatin requires a centromere in *cis*. These observations suggest that the budding yeast centromere and its associated factors together behave as a bidirectional enhancer to increase cohesin binding throughout large pericentric regions in every cell cycle.

**Figure 6:**
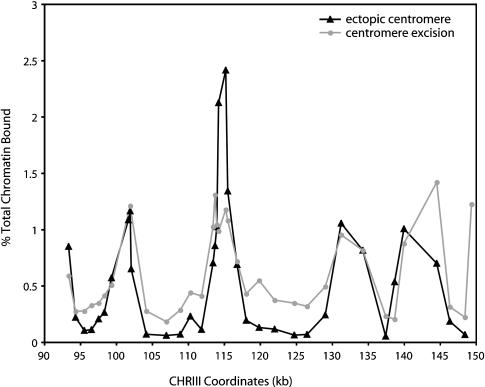
Mcd1p Binding within the Endogenous CHRIII Pericentric Region after Centromere Excision or Centromere Movement The Mcd1p binding profiles in the endogenous CHRIII pericentric region are shown in cells in which the centromere is absent, either because of centromere excision (gray circles, PMY185) or because of the movement of the centromere to an ectopic location on the right arm of CHRIII (black triangles, PMY318). PMY185 and PMY318 are highly related strains; PMY185 was one of the parental strains used to generate PMY318. In the centromere excision strain, Mcd1p binding was examined in the same cell cycle in which the centromere was lost, whereas in the ectopic centromere strain, Mcd1p association was determined many generations after centromere relocation (see text for further discussion). Mcd1p binding data from the centromere excision experiment are the same as those shown in Figure 2B, but are replotted here for clarity. *CEN3* normally occupies the interval between SGD coordinates 114382-114498.

While the removal of the centromere by site-specific recombination greatly reduced the levels of cohesin bound within pericentric DNA, we noted that the levels of binding within the valleys of the pericentric regions remained higher than the valleys observed in centromere-distal regions (see Figure 3B and 3D). This observation suggested that pericentric sequences might contribute to the enhancement of cohesin association independent of the centromere. However, when the CHRIII centromere was moved to an ectopic location on the right arm of the chromosome, the residual levels of Mcd1p binding within the troughs throughout the former pericentric region were further reduced (Figure 6). In fact, this region now more closely resembled typical arm cohesin-association sites, where peaks of binding occur at approximately 10-kb intervals and are separated by regions with minimal or undetectable levels of cohesin association. Thus, this result suggests that cohesin enrichment within pericentric regions is mediated exclusively by the centromere–kinetochore complex and that a centromere-independent pathway does not contribute to the enhanced levels of cohesin binding in pericentric chromatin. The residual levels of cohesin that remain bound in the troughs immediately following centromere excision may reflect a difference in the timing of centromere loss. In the centromere excision experiment, Mcd1p association was examined during mitosis of the same cell cycle in which the centromere was removed, whereas in the ectopic centromere strain, Mcd1p association was examined many generations after centromere removal. Thus, the persistence of cohesin binding during the first cell cycle following centromere excision suggests the existence of an epigenetic component in the recruitment of cohesin to pericentric chromatin. Indeed, possible epigenetic contributions to kinetochore function have been suggested previously in budding yeast (Mythreye and Bloom 2003).

While the 120-bp centromeric DNA was the only conserved DNA sequence present within the excised regions of the two chromosomes, it was possible that some unidentified motif within the excised DNA was instead responsible for the enhancement of pericentric cohesin binding. To rule out this possibility, we tested whether enhancer activity was mediated specifically by the centromere and its associated factors by examining pericentric cohesin binding in cells lacking functional kinetochores. Kinetochore assembly was disrupted using a conditional mutant in the *NDC10* gene. *NDC10* encodes an essential subunit of CBF3, a complex of kinetochore proteins that binds to the conserved centromere DNA element CDEIII and nucleates kinetochore assembly (Goh and Kilmartin 1993; Jiang et al. 1993). At the restrictive temperature of 37 °C, *ndc10-42* mutants assemble a defective kinetochore, and consequently, arrest in G2/M due to the activation of the spindle assembly checkpoint (Doheny et al. 1993). Cultures of the *ndc10-42* mutant and an isogenic wild-type control strain were staged in G1 and then released from the G1 arrest at the restrictive temperature in medium containing nocodazole. After reaching a mitotic arrest, both the mutant and wild-type cultures were processed for ChIP to assess Mcd1p association in pericentric regions.

**Figure 7:**
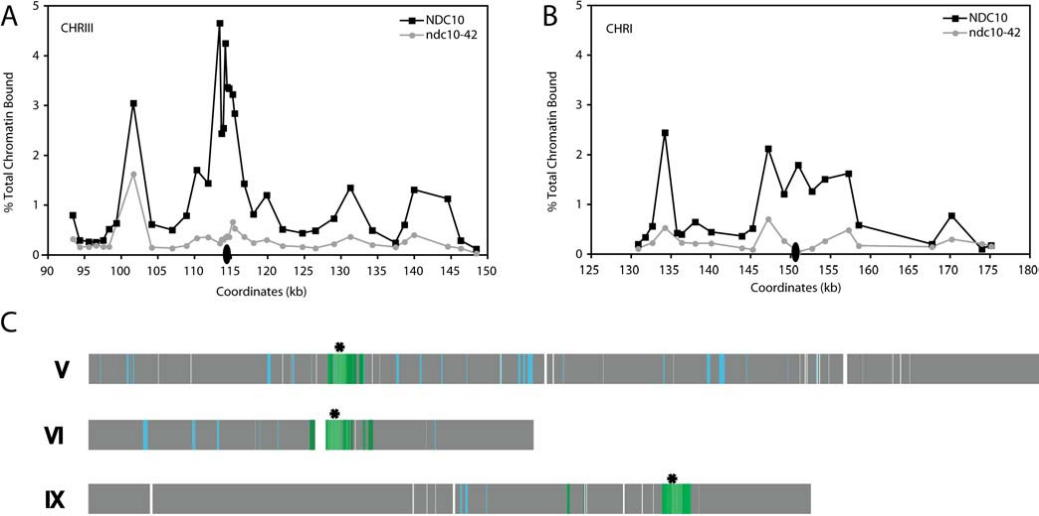
A Functional Centromere–Kinetochore Complex Is Essential for Enhanced Pericentric Cohesin Association Cultures of isogenic wild-type (1846-15A) and *ndc10-42* mutant (1846-15C) cells were arrested in αF at 23 °C and then released into fresh medium containing nocodazole at 37 °C. After the cells arrested in mitosis (approximately 3 h), the cultures were crosslinked with formaldehyde and processed for ChIP using a monoclonal antiserum against epitope-tagged Mcd1p (Mcd1-6HAp) as an indicator of the cohesin complex. The cohesin association profiles in the pericentric regions of CHRIII (A) and CHRI (B) are shown for *NDC10* (black squares) and *ndc10-42* (gray circles) cultures. The positions of the centromeres are indicated by ovals (not drawn to scale). The dashed line in (B) indicates a region containing a Ty element. (C) To identify chromosomal regions depleted for cohesin binding in the absence of a functional kinetochore, the Mcd1p-ChIP-to-input fluorescence ratio obtained for each ORF and intergenic region in genomic microarray analyses of CHRV, CHRVI, and CHRIX in *ndc10-42* cells was divided by the ratio obtained for *NDC10* cells and plotted on a map of the chromosomes. Regions that demonstrated 2.5-fold or greater reduction in Mcd1p binding in the *ndc10-42* mutant are shaded dark green, while lighter green hues represent further fold reductions in Mcd1p binding. Regions where the magnitude of Mcd1p binding was similar in *NDC10* and *ndc10-42* cells are shown in gray. Gaps in the chromosomal maps are genomic regions not represented on the microarrays, while regions shaded blue were present on the arrays but gave no data during hybridizations for reasons described in Materials and Methods. The location of the centromere on each chromosome is indicated by an asterisk.

We observed that Mcd1p binding was reduced 5-fold on average throughout an approximately 50-kb CHRIII pericentric region in the *ndc10-42* cells at the restrictive temperature when compared to the isogenic wild-type control (Figure 7A). In fact, Mcd1p association was reduced throughout the same region that was affected by centromere excision. Similarly, Mcd1p binding throughout an approximately 40-kb pericentric region of CHRI was also reduced approximately 5-fold in the *ndc10-42* mutant when placed at the restrictive temperature (Figure 7B). These results differ from those of a previous study which reported no change in cohesin association at an established endogenous centromere in *ndc10-1* cells (Tanaka et al. 1999). This difference is likely explained by the fact that the previous study examined Mcd1p binding within only one approximately 300-bp centromere-spanning region, whereas we examined Mcd1p association at multiple locations throughout 40-kb pericentric regions. To determine the extent to which kinetochore inactivation affected cohesin association at more centromere-distal locations, we examined global Mcd1p association in the *ndc10-42* mutant at the restrictive temperature using the hybridization of ChIP DNA to microarrays. Consistent with the PCR quantitation of CHRI and CHRIII, we found that the magnitude of Mcd1p binding in the pericentric regions of all chromosomes was indeed reduced in the *ndc10-42* mutant at the restrictive temperature when compared to the isogenic wild-type strain (Figure 7C; for brevity, only CHRV, CHRVI, and CHRX are shown). However, Mcd1p binding at centromere-distal locations was equivalent in wild type and *ndc10-42* mutant cells, indicating that cohesin association in these regions is independent of the centromere–kinetochore complex (Figure 7C). These observations demonstrate that the centromere– kinetochore complex can increase the magnitude of cohesin association bidirectionally from the centromere over regions as large as 25 kb, thereby generating approximately 50-kb pericentric domains that are highly enriched for cohesin binding.

### Centromeric Enhancer Activity Is Context- and Orientation-Independent

Our observations suggested that kinetochores mediate the enhancement of cohesin binding within pericentric regions, but that the distribution of cohesin-binding peaks and valleys is an intrinsic property of the flanking DNA. If correct, we reasoned that the distribution of cohesin within pericentric DNA would be unaltered by the replacement of centromeric DNA with centromeric sequences from a different chromosome. To test this hypothesis directly, we removed *CEN1* and approximately 440 bp of flanking sequences from CHRI and replaced it with approximately 320 bp of pericentric DNA from CHRIII that contained *CEN3*. The patterns of Mcd1p association within the pericentric regions of cells containing the altered or endogenous CHRI were then determined by ChIP in nocodazole-arrested cells.

**Figure 8:**
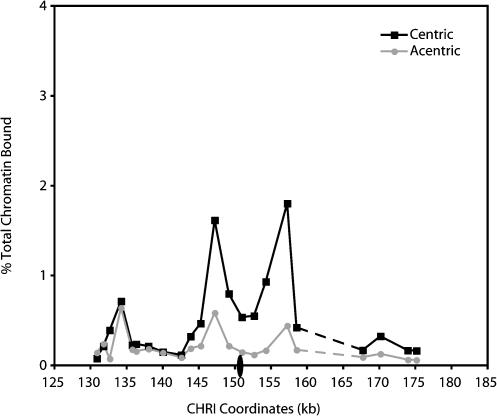
Centromeric Enhancer Activity Is Context and Orientation-Independent Cells containing an endogenous CHRI (1377A1-4B) and those in which *CEN1* was replaced with *CEN3* marked with *URA3* (1824-23B), as described in Materials and Methods, were staged in G1 using αF and then released into fresh medium containing nocodazole. In the case of strain 1824-23B, the G1-arrested culture was split in half, and one half was treated with galactose to induce excision of *CEN3* from CHRI prior to release into medium containing nocodazole. After reaching a mitotic arrest, the cultures were crosslinked with formaldehyde and processed for ChIP using a monoclonal antiserum against epitope-tagged Mcd1p (Mcd1-6HAp). The cohesin association profiles for the modified CHRI with and without *CEN3* are shown (squares and circles, respectively). The position of the centromere is indicated by the oval (not drawn to scale). The dashed line indicates the region containing a Ty element. See Figure 2A for the Mcd1p association profile of the endogenous CHRI.

We observed that both the distribution and the magnitude of Mcd1p binding within the pericentric region of the modified CHRI were similar to those observed on the wild-type CHRI (Figure 8, compare with Figure 3A). Furthermore, the excision of *CEN3* sequences from CHRI using the same experimental regimen described above also resulted in a bidirectional reduction in Mcd1p association in the regions flanking the centromere at both high- and low-affinity regions, as observed previously for CHRIII and CHRXIV (Figure 8). The finding that the magnitude of Mcd1p binding was equivalent within the altered and endogenous CHRI pericentric regions suggested that centromeric enhancers have similar abilities to mediate cohesin association within other pericentric regions. Furthermore, the replacement of *CEN1* with *CEN3* sequences was done in such a way that *CEN3* was present in the opposite orientation with respect to the endogenous centromere, and the context of *CEN3* within the CHRI pericentric region was further modified by the introduction of the *URA3* gene immediately adjacent to the centromere. Thus, these results demonstrate that the centromeric enhancer can function in an altered chromosomal context and that the enhancement of cohesin binding in pericentric DNA is independent of both the primary sequence of the centromere and its orientation with respect to the pericentric sequences.

### Centromeric Enhancer Is Active at an Ectopic Location

The importance of pericentric cohesion in the promotion of sister kinetochore biorientation may have maintained an evolutionary selection for pericentric sequences that favors higher levels of cohesin association. Consequently, these sequences may be particularly susceptible to centromeric enhancer activity. To test whether naive sequences that have never resided near a centromere can also respond to the presence of the centromere–kinetochore complex with increased cohesin association, we determined whether the movement of centromeric DNA to an ectopic location resulted in increased Mcd1p association in the flanking chromatin. In this experiment, Mcd1p association was examined within an approximately 37-kb region on the right arm of CHRIII spanning SGD coordinates 242–279 kb after the insertion of *CEN6* DNA at SGD coordinate 260 kb. Endogenous *CEN3* sequences were removed and the new centromere was inserted concurrently to prevent the production of a dicentric CHRIII (Materials and Methods). Cultures of cells containing the ectopic centromere or an isogenic control strain with the centromere at its endogenous location were staged in G1, and then released into fresh media containing nocodazole to arrest the cells in mitosis. Once the cells were arrested, they were processed for ChIP.

**Figure 9:**
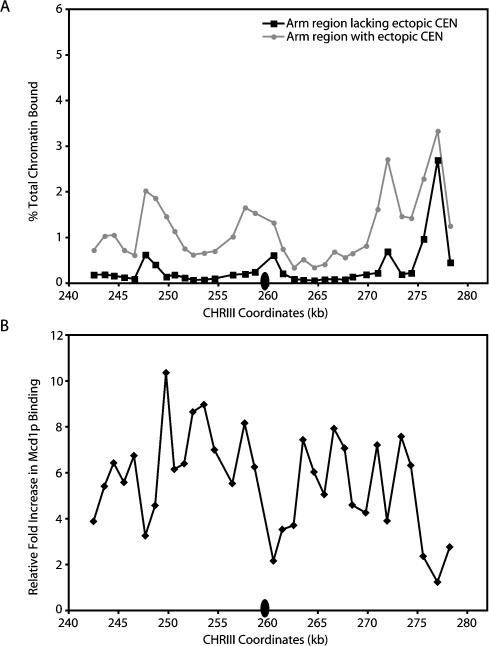
The Centromeric Enhancer Is Active at an Ectopic Location The endogenous centromere on CHRIII was removed, and *CEN6* was inserted at an ectopic location (SGD coordinate approximately 260 kb), producing yeast strain PMY318, as described in Materials and Methods. Cells containing the ectopic centromere and isogenic wild-type cells (1829-15B) were staged in G1 using αF, and then released into fresh medium containing nocodazole to arrest cells in mitosis. Cells were then fixed in formaldehyde and processed for ChIP using epitope-tagged Mcd1-6HAp as a marker for the cohesin complex. (A) The Mcd1p binding profiles at the ectopic location on endogenous CHRIII (black squares) and in the presence of the ectopic centromere (gray circles) are shown. The location of the ectopic centromere is indicated by the black oval. (B) The levels of Mcd1p binding in the region flanking the ectopically placed centromere were divided by those observed in the isogenic wild-type control strain to determine the fold increases in Mcd1p binding in the presence of the centromere. Data are plotted as a function of the SGD coordinates for this region.

Quantitation of the levels of Mcd1p-associated sequences in the wild-type cells revealed a major peak of Mcd1p binding at SGD coordinate approximately 277 kb and three minor peaks at 248 kb, 261 kb, and 272 kb (Figure 9A). In cells containing the ectopically placed centromere, the peaks of Mcd1p binding occurred at the same locations as those observed in the wild-type cells, but the magnitude of binding was significantly higher, most notably within the peaks at 248 kb and 273 kb. Moreover, the levels of Mcd1p binding in the troughs proximal to the ectopic centromere were also dramatically elevated. Indeed, when the fold increases in cohesin binding flanking the ectopic centromere were plotted, the analysis revealed that similar levels of enhancement are reached throughout the approximately 37-kb region examined (median fold increase of 6.0 ± 2.1), with the majority of the increase occurring in the trough regions (Figure 9B). Thus, the insertion of the centromeric enhancer resulted in the generation of a cohesin domain (≥37 kb) similar in size to that mediated by an endogenous centromere–kinetochore complex. In addition, cohesin binding at the ectopic location was not random, but instead, appeared to amplify the intrinsic pattern of association. Taken together, these observations indicate that the ability of the centromere to enhance cohesin binding in flanking DNA is not limited to endogenous pericentric sequences.

## Discussion

In this report we show that the approximately 120-bp point centromere of budding yeast increases the magnitude of cohesin association within large approximately 20–50-kb pericentric regions. The budding yeast centromere–kinetochore complex generates a specialized chromatin structure, consisting of an approximately 250-bp nuclease-resistant region flanked by several positioned nucleosomes that together span approximately 3 kb (Bloom and Carbon 1982). Thus, the centromere-flanking cohesin domains are approximately 80–200 times larger than the nuclease-resistant region of the kinetochore. The ability of kinetochores to mediate increased cohesin association over large domains was not limited to endogenous pericentric sequences, but also occurred when the centromere was moved to an ectopic location. Although rare, other examples exist in which *cis* DNA elements have been shown to mediate the generation of large chromosomal domains, namely telomeric silencing and X chromosome inactivation (Renauld et al. 1993; Hecht et al. 1996; Lee et al. 1999). Finally, the kinetochore enhanced cohesin association in pericentric regions only in *cis* and in an orientation-independent manner. Thus, the kinetochore functions as an enhancer of pericentric cohesin binding in addition to mediating the attachment to the mitotic spindle.

The kinetochore-dependent generation of these extended pericentric cohesin domains may be the consequence of kinetochore-mediated de novo loading of cohesin, or, alternatively, the domains may reflect a role for the kinetochore in maintaining or protecting cohesin association in centromere-flanking regions. Evidence in support of both models exists. Cohesin association in the centromere-flanking region of a minichromosome was reduced upon centromere excision in M phase–arrested cells (Megee et al. 1999). In addition, high levels of cohesin association were observed in the CHRIII pericentric region of cells arrested in S phase or mitosis, while cohesin binding along the arms was lower in mitotically arrested cells compared to cells arrested in S phase (Blat and Kleckner 1999). These observations are consistent with a role for the kinetochore in maintaining cohesin association in pericentric regions. However, the elevated levels of pericentric cohesin binding were not identical in the S phase– and M phase–arrested cells, but were in fact higher in the mitotically arrested cells (Blat and Kleckner 1999). Furthermore, we have found that the magnitude of cohesin binding within pericentric regions of mitotically arrested cells increases in response to environmental cues (P. Megee, unpublished data). These observations are consistent with the de novo loading of cohesins by the centromere–kinetochore complex. Thus, the kinetochore may mediate increased cohesin binding by multiple pathways.

Our finding that kinetochores mediate cohesin binding throughout large approximately 50-kb pericentric domains may potentially reconcile seemingly paradoxical observations concerning the enrichment of cohesin in pericentric chromatin and the transient loss of cohesion between sister chromatids in these regions. Despite the presence of these large pericentric cohesin domains, sister chromatids undergo transient separations within pericentric regions shortly after the formation of bipolar spindle microtubule attachments (Goshima and Yanagida 2000; He et al. 2000; Tanaka et al. 2000). However, the extent of sister chromatid separation observed in these studies is consistent with the deformation of only approximately 20 kb of pericentric DNA (He et al. 2000, 2001). Thus, our results indicate that pericentric cohesin domains extend well beyond the approximately 20 kb of chromatin undergoing significant microtubule-dependent stretching. These observations suggest that pericentric cohesion may play dual roles in the maintenance of genomic integrity. First, cohesin bound immediately adjacent to the kinetochore may sterically constrain the kinetochores on paired sister chromatids to face opposite poles, thereby facilitating the establishment of chromosome biorientation. Once biorientation is achieved, the poleward forces imposed by the microtubule attachments disrupt cohesin binding within approximately 20-kb pericentric regions, giving rise to transient sister chromatid separation. Because the kinetochore-mediated pericentric cohesin domain extends beyond the region of stretched chromatin, this domain may provide the resistance that is required for the production of tension between sister kinetochores (Skibbens et al. 1995). This tension is thought to promote the stability of kinetochore–microtubule attachments (Nicklas and Ward 1994).

Our results also demonstrate that the distribution of cohesin within each pericentric domain differs between chromosomes and that the observed pattern is unrelated to the specific centromeric DNA sequence present on the chromosome. Instead, the distribution of cohesin in either endogenous or ectopic centromere-proximal locations is specified by an intrinsic property of the chromatin. Furthermore, although the ability of the centromeric enhancer to mediate cohesin binding extended over large domains, the loss of the kinetochore did not affect cohesin binding in centromere-distal regions (this study). This observation is consistent with two alternative explanations. First, the centromeric enhancer may form a gradient of activity that dissipates with distance from the kinetochore. This model is unlikely, however, since the fold increases in cohesin association adjacent to the ectopic centromere were similar throughout the flanking approximately 37-kb region. Alternatively, the length of pericentric chromatin that can be influenced by the centromeric enhancer may be constrained by *cis* factors, such as boundary elements or sequences nonpermissive for cohesin binding. Such a model is supported by the relatively small pericentric cohesin domain present on minichromosomes, where local sequence context was suggested to affect cohesin binding adjacent to the centromere (Megee et al. 1999). Interestingly, during meiosis cohesins are removed from chromosome arms but remain bound in the large pericentric regions (Klein et al. 1999; Watanabe and Nurse 1999). It is possible that the boundaries that dictate the pericentric region and constrain the centromeric enhancer are the same that modulate cohesin binding during meiosis. Moreover, since the distribution of cohesin association in pericentric regions appears to be unique to each chromosome, this variability in cohesin distribution may provide a basis for the range of nondisjunction frequencies associated with different chromosomes within an organism (Campbell et al. 1981).

In this report we have provided insights into the control of cohesin binding within pericentric chromatin in budding yeast. We have demonstrated that the centromere–kinetochore complex behaves as an enhancer for cohesin association in pericentric chromatin and appears to be largely responsible for the increased levels of pericentric cohesin association at both endogenous and ectopic locations. Presumably, the enhancer functions by activating a *trans* factor that either recruits cohesin to pericentric chromatin or maintains high levels of pericentric cohesin binding. One candidate for such a *trans* factor could be a histone-modifying enzyme. The centromeric enhancer would augment the recruitment of the histone-modifying enzyme and enhance cohesin association. Indeed, results from S. pombe indicate that a histone methyltransferase is targeted to centromere-proximal heterochromatin, and this modification is important for cohesin recruitment (Bernard et al. 2001; Nonaka et al. 2002). Since budding yeast lacks centromere-proximal heterochromatin, the targeting method for pericentric cohesin recruitment is likely to be different. However, since pericentric cohesin domains are highly conserved, we suspect that the enhancer activity of the kinetochore may well be ubiquitous in all eukaryotes.

Our results suggest that the coordination of microtubule attachment and pericentric cohesin recruitment by the budding yeast kinetochore generates an autonomous segregation unit that ensures sister kinetochore biorientation and, consequently, the maintenance of genomic integrity. The integration of these activities by the kinetochore was likely key for the first identification of budding yeast centromeric DNA, since microtubule attachment in the absence of pericentric cohesion is unlikely to have increased the mitotic stability of minichromosomes, the assay used for centromere DNA identification (Clarke and Carbon 1980). Similarly, the coordination of cohesion and microtubule attachment by the kinetochore may also explain how neocentromeres in humans acquire full chromosome segregation capabilities in the absence of flanking repetitive DNA or cytologically detectable levels of pericentric heterochromatin (Aagaard et al. 2000; Saffery et al. 2000; Amor and Choo 2002). Our observations suggest that kinetochore-mediated cohesin recruitment may compensate for the lack of heterochromatin-dependent cohesin recruitment, thereby promoting biorientation of sister kinetochores. Moreover, the ability of a neocentromere to generate new domains of cohesin binding is likely to have been instrumental for chromosome evolution by allowing some degree of flexibility for chromosomal rearrangements.

## Materials and Methods

### 

#### Yeast cell culture

The genotypes of the yeast strains used in this study are listed in Table 1. Cultures used for ChIP were synchronized in G1 using α-factor (αF) mating pheromone at final concentrations of 3 μM and 15 nM for *BAR1* wild-type and mutant strains, respectively. Cells were then released from the G1 arrest by two washes in the appropriate growth medium containing 0.1 mg/ml Pronase (Sigma, St. Louis, Missouri, United States) to proteolyze the αF. Cells were then allowed to grow in medium containing 0.1 mg/ml Pronase and then arrested in mitosis using either 15 μg/ml nocodazole (Sigma) resuspended in 1% DMSO, or a temperature-sensitive *cdc16* mutation, as indicated. Mitotic arrest, as determined by a large-budded cell morphology, was generally reached after 2.5–3 h of growth. The R-site-specific recombinase from Zygosaccharomyces rouxii used for centromere excision events was induced in G1 cells by the addition of galactose (2% final concentration) to rich medium containing 2% raffinose.

**Table 1:**
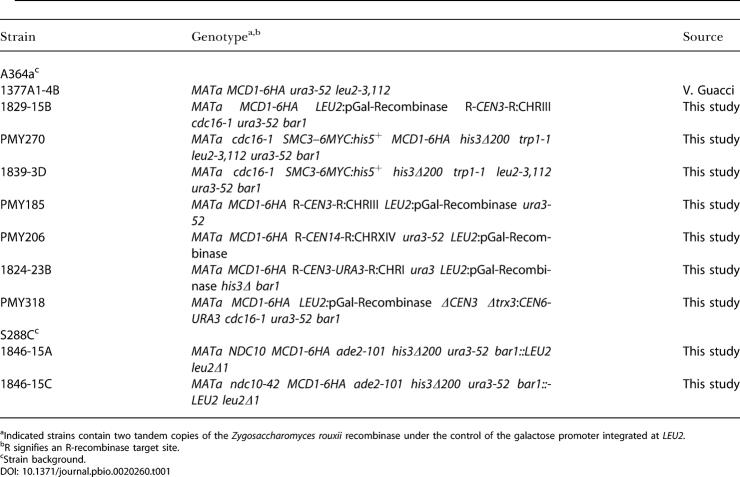
Saccharomyces cerevisiae Strains

^a^Indicated strains contain two tandem copies of the Zygosaccharomyces rouxii recombinase under the control of the galactose promoter integrated at *LEU2.*

^b^R signifies an R-recombinase target site

^c^Strain background

#### Centromere excision and strain construction

Centromeres were excised from endogenous chromosomes by site-specific recombination events using a galactose-inducible R recombinase from Zygosaccharomyces rouxii (Matsuzaki et al. 1990). A 320-bp BamHI *CEN3* fragment was inserted between head-to-tail-oriented recombination target sites, as described previously (Megee and Koshland 1999). *URA3* was subcloned adjacent to the *CEN3* centromere cassette outside the recombination target sites, and this construct replaced the endogenous centromere on CHRIII by a one-step replacement (Rothstein 1983), selecting for uracil prototrophs. To restore the chromosomal context of the centromere on CHRIII, the one-step replacement was then repeated with a centromere cassette lacking *URA3,* and transformants were grown on 5-fluoroorotic acid to screen for the loss of *URA3*. The constructs were then confirmed by Southern analysis of genomic DNA. Similarly, a 334-bp PCR fragment (SGD coordinates 628717-629051) containing *CEN14* and approximately 200 bp of flanking DNA was inserted between head-to-tail-oriented recombination target sites. A *URA3*-marked version of this construct was used to replace the endogenous centromere on CHRXIV by one-step gene replacement, and the chromosomal context of *CEN14* was then restored using the same strategy outlined above for *CEN3*. The construction of the *CEN3* replacement of *CEN1* DNA was performed similarly, except that *URA3* was placed adjacent to the centromere between the recombination target sites. Replacement of *CEN1* sequences with the *CEN3-URA3* cassette resulted in the deletion of 558 bp containing *CEN1* (CHRI, SDG coordinates 151263-151820), and was confirmed by Southern analysis of genomic DNA (unpublished data). To obtain PMY318, a strain having the centromere at an ectopic location on CHRIII, strain 1829-15B was transformed with DNA encoding a *CEN6-URA3* cassette flanked by regions homologous to the *TRX3* locus. The *TRX3* ORF and a small amount of flanking sequences, encompassing SGD coordinates 259521-260061, were deleted by the integration of the *CEN6-URA3* cassette. Prior to transformation, 1829–15B cells were grown in rich medium containing raffinose, and then plated on medium containing galactose to induce excision of endogenous *CEN3* and approximately 200 bp of the flanking region by site-specific recombination, as described above.

#### ChIP

ChIP was performed as described (Megee et al. 1999). A detailed protocol is available at http://www.uchsc.edu/sm/bbgn/megee.html (see also Protocol S1 in the accompanying paper by Glynn et al. [2004]). Immunoprecipitations were done using 12CA5 anti-HA antibody (Roche, Basel, Switzerland), A-14 anti-Myc antibody (Santa Cruz Biotechnology, Santa Cruz, California, United States), or rabbit polyclonal anti-Mif2p antibody (Meluh and Koshland 1997), as indicated. Immunoprecipitations of crosslinked chromatin prepared from strains lacking epitope-tagged versions of cohesin subunits did not precipitate centromeric DNA, as demonstrated previously (Megee et al. 1999). In addition, mock immunoprecipitations were performed to exclude the possibility that centromere-flanking chromatin was nonspecifically enriched in the immunoprecipitates obtained from epitope-tagged strains. We observed that centromeric sequences were enriched over those observed in the mock immunoprecipitations at least 500-fold and 11-fold using HA-tagged or Myc-tagged cohesin subunits, respectively. This difference likely reflects the observation that for any cohesin-associated region, a larger percentage of total chromatin is precipitated with the HA epitope tag than with the Myc tag (Mcd1-6HAp compared to Mcd1-18Mycp) (P. Megee, unpublished data). Lastly, analysis of the input DNA showed that our samples were routinely sheared to an average size of 500 bp, with a range of 200–1,000 bp.

In all experiments, duplicate immunoprecipitations were performed for cohesin subunits and subjected to a preliminary PCR analysis using centromere-specific or -proximal oligonucleotide primer pairs. The duplicates had values that were routinely within 10% of one another, and, thus, one sample was chosen randomly for further analysis. The linearity of PCR under our experimental conditions was tested empirically. Briefly, input DNA was diluted 90-fold relative to immunoprecipitated DNA before PCR analysis. Increasing amounts of input DNA were then used to program PCR reactions, and the resulting products were quantitated to determine whether the amount of product responded linearly to the levels of input DNA (unpublished data; Megee et al. 1999). This relationship was determined empirically in experiments initiated with approximately 1.65 × 10^9^ cells, and all subsequent experiments were performed with the same cell number to maintain linearity of PCR.

PCR fragments were separated on 2.5% NuSieve (Cambrex, East Rutherford, New Jersey, United States) gels containing 0.15 μg/ml ethidium bromide. Digital images of ethidium bromide–stained gels were quantitated using ImageQuant software (Molecular Dynamics, Sunnyvale, California, United States). The details of oligonucleotide primer pairs used for PCR analyses are available upon request. In general, PCR primer pairs amplified approximately 300-bp sequences and were separated from neighboring pairs by an average of approximately 1.5 kb. In the CHRIII pericentric region, the mean size of the 37 PCR products used in the ChIP analysis was 304 ± 68 bp. For CHRXIV, the mean size of the 41 PCR products used in the analysis was 309 ± 36 bp. In the CHRI pericentric region, the mean size of the 22 PCR products used in the analysis was 306 ± 31 bp. ChIP experiments were performed at least twice, and representative data from one experiment are presented.

#### Microarray analyses of ChIP DNA

Preparation of Cy5- and Cy3-labeled DNA, hybridization, and analysis were performed as previously described (Gerton et al. 2000). Briefly, the recovered ChIP DNA was randomly PCR amplified in the presence of amino-allyl dUTP (Bohlander et al. 1992; Gerton et al. 2000; see also Protocol S2 in the accompanying paper by Glynn et al. [2004]), which was then coupled to a fluorescent dye (e.g., Cy5) and competitively hybridized to a polyL-lysine-coated spotted glass DNA microarray in the presence of total genomic DNA similarly labeled with a second fluorescent dye (e.g., Cy3). Hybridizations were performed at 63 °C overnight under standard conditions and slides were washed successively with 0.6X SSC/0.03% SDS and then 0.06X SSC prior to scanning (see also http://microarrays.org). The fluorescence in each spot on the microarray was detected using an Axon 4000B laser scanner/detector (Axon Instruments, Union City, California, United States), and the ratio of the two signals was determined using GenePix 4.0 software (Axon Instruments). This ratio indicates the enrichment for a given sequence in the ChIP. The AMAD database was used to normalize and store microarray data. The microarrays used in this study contained all the ORFs and intergenic regions in the yeast genome as individual spots (Iyer et al. 2001). For each experimental condition, a minimum of two hybridizations from two independent immunoprecipitations was performed. Data were normalized and then filtered in the following ways: the spot intensity was required to be 200 or greater; flagged spots were not used; and spots were required to have a correlation coefficient of 0.5 or greater. For analysis purposes, any feature with less than two measurements was excluded, and excluded regions are colored blue in the maps of cohesin binding. The raw data and datasets for the microarrays presented herein are provided as Datasets S1–S22, and can also be viewed at http://research.stowers-institute.org/jeg/2004/cohesin/index.html.

## Supporting Information

Datasets S5–S22 correspond to the individual GenePix results (GPR) files for each array performed. For each dataset, the Cy3 and Cy5 channel samples and the method of mitotic arrest are listed.

Dataset S1 Dataset for Smc3-6Myc ChIPs Performed in *cdc16*-Arrested Cells File cdc16_Smc3-6Myc_A364a.
(701 KB TXT).

Dataset S2 Dataset for Mcd1-6HA ChIPs Performed in *cdc16*-Arrested Cells File cdc16_Mcd1-6HA_A364a.
(957 KB TXT).

Dataset S3 Dataset for Mcd1-6HA ChIPs Performed in Nocodazole-Arrested *NDC10* Cells File Mcd1-6HA_S288c_NZ.
(449 KB TXT).

Dataset S4 Dataset for Mcd1-6HA ChIPs Performed in Nocodazole-Arrested *ndc10-42* Cells File Mcd1-6HA_ncd10-42_S288c_NZ.
(418 KB TXT).

Dataset S5 SIMRUP2_147 Cy3 = *cdc16*-ts Mcd1-6HA ChIP in A364a; Cy5 = genomic DNA.
(4.6 MB XLS).

Dataset S6 SIMRUP2_170 Cy3 = *cdc16*-ts Mcd1-6HA ChIP in A364a; Cy5 = genomic DNA.
(4.6 MB XLS).

Dataset S7 SIMRUP2_171 Cy3 = *cdc16*-ts Mcd1-6HA ChIP in A364a; Cy5 = genomic DNA.
(4.6 MB XLS).

Dataset S8 SIMRUP2_178 Cy3 = genomic DNA; Cy5 = *cdc16*-ts Mcd1-6HA ChIP in A364a.
(4.6 MB XLS).

Dataset S9 SIMRUP2_180 Cy3 = genomic DNA; Cy5 = *cdc16*-ts Mcd1-6HA ChIP in A364a.
(4.6 MB XLS).

Dataset S10 SIMRUP2_183 Cy3 = Mcd1-6HA ChIP in nocodazole-arrested S288C background; Cy5 = genomic DNA.
(4.6 MB XLS).

Dataset S11 SIMRUP2_184 Cy3 = *ndc10-42* Mcd1-6HA ChIP in nocodazole-arrested S288C background; Cy5 = genomic DNA.
(4.6 MB XLS).

Dataset S12 SIMRUP2_185 Cy3 = Mcd1-6HA ChIP in nocodazole-arrested S288C background; Cy5 = genomic DNA.
(4.6 MB XLS).

Dataset S13 SIMRUP2_187 Cy3 = *cdc16*-ts Smc3-6Myc ChIP in A364a; Cy5 = genomic DNA.
(4.6 MB XLS).

Dataset S14 SIMRUP2_190 Cy3 = ChIP of Mcd1-6HA in *cdc16*-ts Smc3-6Myc Mcd1-6HA A364a strain; Cy5 = genomic DNA.
(4.6 MB XLS).

Dataset S15 SIMRUP2_191 Cy3 = ChIP of SMC3-6Myc in *cdc16*-ts SMC3-6Myc MCD1-6HA A364a strain; Cy5 = genomic DNA.
(4.6 MB XLS).

Dataset S16 SIMRUP2_226 Cy3 = genomic DNA; Cy5 = *cdc16*-ts Smc3-6Myc ChIP in A364a.
(4.6 MB XLS).

Dataset S17 SIMRUP2_228 Cy3 = genomic DNA; Cy5 = Mcd1-6HA ChIP in nocodazole-arrested S288C background.
(4.6 MB XLS).

Dataset S18 SIMRUP2_229 Cy3 = genomic DNA; Cy5 = *ndc10-42* Mcd1-6HA ChIP in nocodazole-arrested S288C background.
(4.6 MB XLS).

Dataset S19 SIMRUP2_230 Cy3 = genomic DNA; Cy5 = Mcd1-6HA ChIP in nocodazole-arrested S288C background.
(4.6 MB XLS).

Dataset S20 SIMRUP2_231 Cy3 = genomic DNA; Cy5 = *ndc10-42* Mcd1-6HA ChIP in nocodazole-arrested S288C background.
(4.6 MB XLS).

Dataset S21 SIMRUP2_254 Cy3 = genomic DNA; Cy5 = ChIP of SMC3-6Myc in *cdc16*-ts Smc3-6Myc Mcd1-6HA A364a strain.
(4.6 MB XLS).

Dataset S22 UP6_205 Cy3 = genomic DNA; Cy5 = Mcd1-6HA ChIP in nocodazole-arrested S288C background.
(4.4 MB XLS).

### Accession Numbers

The *Saccharomyces* Genome Database (http://www.yeastgenome.org/) accession numbers for the genes and gene products discussed in this paper are Irr1p/Scc3p (SGDID S0001288), Mcd1/Scc1p (SGDID S0002161), Mif2p (SGDID S0001572), Pds5p (SGDID S0004681), Smc1p (SGDID S0001886), and Smc3p (SGDID S0003610). The S. pombe Genome Project (http://www.sanger.ac.uk/Projects/S_pombe/) accession number for Swi6 is SPAC664.01c.

## Abbreviations

αF: α-factor mating pheromone
ChIP: chromatin immunoprecipitation
CHR: chromosome
ORF: open reading frame
R:G: red-to-green
SGD: *Saccharomyces* Genome Database
